# The Physic Bottle

**Published:** 1887-12-10

**Authors:** 


### THE DOCTOR'S PULPIT.

### THE PHYSIC BOTTLE.

SOME few years ago a young Lancashire medical man had arranged to go with two or three curates and a few ladies for a trip to Bolton Woods. Before starting he made up a gallon of coloured and slightly-flavoured mixture, and instructed a friend who was left behind that if any patients sent for medicine in his absence their "bottles" were to be filled out of this common stock. No matter what their ailments, they were all to have exactly the same physic.

People who have no faith in "drugs," and others who regard doctors as "humbugs," will find in this little story strong evidence of the justice of their sentiments. Yet the Lancashire doctor was not at all a "humbug," nor did he disbelieve in physic. He had a large *clièntele* of working people—mill-hands and colliers. None of them happened to be at all seriously ill at the moment; a dozen at least were sure to send for physic—ten of whom did not really require it, and the other two could very well afford to wait until next day. The doctor was a perfect slave to his patients, and needed a holiday, and so he did a little casuistical practice, and boldly took the breathing time he required. If he had left no physic behind, the patients would have had to go away empty-handed, and would have thought themselves ill-used. Having, however, their dearly-beloved "bottles" filled to the neck with a prettily-coloured and nicely-flavoured fluid, with a clean label instructing them to take "the mixture as before," they were perfectly happy.

The story well illustrates the position occupied by the *physic bottle* in the medical practice of the day. A very large number of persons hold the idea that when they are ill the beginning, the middle, and the end of treatment is a bottle or bottles of medicine. This feeling is by no means confined to the poor. At the present time it shows itself in a way which proves that many of the more educated have precisely the same views about the matter. For example, A says to B, "You are looking out of sorts; why don't you see Dr. ——?" mentioning the family attendant. "Oh," says B, "I never bother with him; he sees the wife and children, but when I want anything for myself I go to Dr. ——, of Candy-sugar Square; he gives me a prescription which soon makes me alright." This man, too, though he can pay a two or three guinea fee, and presumably, therefore, has some education, considers the "physic bottle" the sheet anchor of practice.

It is really a pity—a serious pity—that this settled belief, which is almost universal, should have taken such firm hold of the public mind. The feeling in favour of the physic bottle is akin to the old superstitious regard for charms and incantations. There is an ineradicable hankering in the heart of man for the rapid, the sensational, the wonderful. The crowds who gape and stare at the quack doctor on a Saturday night, with his pill that cures every mortal ill, earthquakes included, are doing exactly what most of the inhabitants of the West End squares would do if they could gratify their instincts in a drawing-room and consistently with "good form." This general sentiment is a strong barrier to recovery in the time of sickness, and an equally potent factor in the production of many varieties of ill-health. Men do not recognise that a great part of medical practice has to be done by themselves when they are slightly ill; and by the nursing attendant when they are more seriously attacked; and that the "physic" is but one of the many details which must engage the attention of patient, physician, and nurse. A physician, be he never so skilful, cannot do that duty which properly appertains to his patients. It is with the doctor as it is with the clergyman: the latter may be the ablest reasoner and the most eloquent declaimer in the world, and yet if his hearers will make no resolute efforts on their own behalf the only result of his preaching will be to wear out his own heart and to leave his congregation no more self-denying, no more virtuous, no more truly noble than before. It is exactly so with the medical man. His *physic* is comparatively a trifle in many cases; his *advice* is worth more than gold. But whilst the majority of people will take the physic, it is only here and there one who with all his heart believes in and tries to carry out the advice. The true physician, indeed, has much the same feeling towards his patients as the nobler clergymen have towards their congregations and parishes. He puts his whole heart into his labours, and is never so satisfied as when he restores his patients promptly and perfectly to health.

All men ought to be made to understand and realise that in every department of life self-help and self-reliance are of the greatest value. There must be specialists like doctors, lawyers, preachers, and all classes of teachers, leaders, and business men. But no one should give up his reason and his personal care of his own affairs because he is obliged to seek the aid of such specialists. How very few are the conditions in which a man can afford to say, "My personal and individual responsibilities cease here. I must now abandon myself entirely to the judgment and unchecked control of another."

"Self-help" is one of the first principles of physical well-being, as it is also of worldly prosperity and of moral health. A man should make it his duty, as it is clearly his interest, to understand as much about his bodily structure and what is called his "constitution" as he possibly can. His natural physical and mental powers and his health are generally the only weapons he has to fight the battle of life with. Is it not more than important, then, that these weapons should be constantly kept in a high state of efficiency and readiness? And who in all the world can have the same immediate and urgent interest in these things as a man himself? By all means let a person consult the doctor as often as he pleases, and let him take, methodically and conscientiously, all the medicine an honest and intelligent practitioner prescribes. Many medicines are absolutely essential to recovery; and none should ever be despised when prescribed by a trustworthy doctor. But we appeal to the experience of every man and woman who has ever consulted a medical practititioner: Did that practitioner in a single instance order physic merely, without saying a word about diet, exercise, habits, and so forth? Is it not true that in many instances, especially when the ailments have been such as to permit of a continuance of the daily occupation, he gave most minute instructions on these points and on many others, such as the avoidance or diminution of stimulants, early rising and early retiring, the moderation of labour, and so on, finishing up the consultation by prescribing a certain mixture? And must it not be confessed that in many cases the physic alone was taken, all the rest of the consultation being entirely thrown away and forgotten, as if it were not of the slightest moment? Now that is absolute stupidity. Sometimes, it is admitted, the "physic bottle" properly occupies the place of the roast beef in the medical meal; but even then how many other things are required to make the meal complete! Frequently, however, it only plays the part of the pudding or the celery, and not seldom it is a mere condiment, like anchovy sauce or even common salt.

We claim for the intelligent and conscientious doctor more docility and attention on the part of the patient; a greater desire to understand what he has to teach; a more earnest effort to get at his mind. It is a mind worth getting at; and the only reason why he does not try to impress his deeper experiences and convictions upon his patients is that they do not give themselves up earnestly and with a will to learn thoroughly all the lore he so much desires to impart. How much more service every earnest man could render to the world if those who seek his counsel and aid would try with all their hearts to practice perfectly what he preaches sincerely! A single grain of intelligent conviction on the patient's part is worth a great many physic bottles.

